# Trial of transcutaneous electrical acupoint stimulation in laryngopharyngeal reflux disease: study protocol for a randomized controlled trial

**DOI:** 10.1186/s13063-022-06193-0

**Published:** 2022-04-08

**Authors:** Hailong Shen, Yanxun Han, Di Wu, Lihong Hu, Yunxia Ma, Feihu Wu, Ye Tao, Yehai Liu

**Affiliations:** 1 Department of Otolaryngology-Head & Neck Surgery, The First Affiliated Hospital of Anhui Medical University, No. 81 Meishan Road, Shushan District, Hefei, Anhui 230022 People’s Republic of China; 2 Department of Otolaryngology, The First Affiliated Hospital of Anhui University of Chinese Medicine, Hefei, Anhui 230031 People’s Republic of China

**Keywords:** Transcutaneous electrical acupoint stimulation (TEAS), Laryngopharyngeal reflux disease (LPRD), Proton pump inhibitors (PPIs), Treatment

## Abstract

**Background:**

Patients with persistent globus sensation, throat clearing, chronic cough, hoarseness, and other throat symptoms account for a large proportion of patients in ears, nose, and throat clinics. Laryngopharyngeal reflux disease (LPRD) is increasingly valued by otolaryngologists. Transcutaneous electrical acupoint stimulation (TEAS) is possibly a new method for the treatment of LPRD. This trial aims to determine whether TEAS combined with proton pump inhibitor (PPI) is better than PPI alone in the treatment of LPRD.

**Methods:**

This prospective randomized controlled trial will be implemented in a tertiary hospital in China. Seventy patients diagnosed with LPRD will be randomly assigned to the TEAS + PPI group (intervention group) or PPI group (control group), at a ratio of 1:1. In addition to using PPI, the intervention group will receive TEAS at four groups of acupoints, and each group will be treated for 15 min, once for 60 min, five times a week, for 12 weeks, 60 times. The main outcome will be changes in the Reflux Symptom Index scores at 4, 12, and 24 weeks after treatment. The secondary outcomes will include changes in the reflux finding score, Laryngopharyngeal Reflux-Health-related Quality of Life score, and throat pain visual analog scale score.

**Discussion:**

This trial will explore the feasibility of TEAS combined with PPI for the treatment of LPRD and provide potential evidence for its effectiveness and safety. The results of this study will be published in a peer-reviewed journal.

**Trial registration:**

Chinese Clinical Trial Registry ChiCTR2100046755. Registered on May 28, 2021.

**Supplementary Information:**

The online version contains supplementary material available at 10.1186/s13063-022-06193-0.

## Background

According to the 2002 statement of the American Academy of Otorhinolaryngology Head and Neck Surgery, laryngopharyngeal reflux (LPR) refers to the backflow of stomach contents into the laryngopharynx [1]. A recent review provided a more complete definition [2], which is defined as a type of upper aerodigestive tract inflammation related to the direct or indirect effects of gastroduodenal reflux, which may cause morphological changes. This definition considers that LPR stimulation caused by pepsin, bile salts, and other gastroduodenal proteins not only involves the mucosa of the laryngopharynx but also should include all mucosa of the upper gastrointestinal and respiratory tracts. Laryngopharyngeal reflux disease (LPRD) refers to a series of symptoms and signs experienced in the throat, middle ear, nasal cavity, trachea, and lung caused by LPR. There are two possible pathophysiological mechanisms of LPR: throat injury can be caused by direct contact and stimulation of reflux contents [3]. The other is that gastroduodenal contents can also stimulate the distal esophagus, stimulate chemoreceptors, and cause reflex cough, obstruction, and hypersecretion of the laryngopharyngeal mucosa [4–6]. The accumulation of viscous mucus can cause throat discomfort, such as postnasal drip, globus sensation, throat clearing, and coughing.

Xiao and Chen et al. reported that based on the Reflux Symptom Index (RSI) assessment, the prevalence of LPRD in China is approximately 10.15–18.8% [7, 8]. Similarly, two experts estimate that 5% of Greeks and 30% of British people experience LPR [9, 10]. Moreover, approximately half of patients with laryngeal and voice disorders have reflux factors [11]. Altman et al.’s study showed that between 1990 and 2001, the number of patients attending ears, nose, and throat (ENT) clinics due to LPR increased by 500% [12].

A study in the USA found that the cost of treating extraesophageal reflux symptoms in 281 patients was more than five times the estimated cost of treating patients with traditional gastroesophageal reflux disease (GORD) symptoms and that more than 50% of these costs were attributable to the use of proton pump inhibitors (PPIs). Therefore, LPR not only causes patient discomfort but also places a heavy economic burden on the US medical system [13].

PPI has become the first choice for the treatment of LPRD, although there is no particularly solid data support. Two recent meta-analyses based on randomized controlled trials have shown that PPI drugs have either no significant benefit or only a slight advantage over placebo [14, 15]. PPI treatment of LPR can only reduce the acidic components in the reflux but cannot completely prevent it; moreover, other more destructive reflux components, such as pepsin and bile acid, can survive even without strong acids. Therefore, it is of great significance to explore more effective treatments for LPRD.

Acupuncture is a common method of traditional Chinese medicine treatment. Studies have shown that acupuncture is used for the treatment of LPRD and has achieved good results. Transcutaneous electrical acupoint stimulation (TEAS) is a noninvasive, safe, and comfortable treatment that combines transcutaneous electrical nerve stimulation and acupoint stimulation. It is clinically used to replace electric and manual acupuncture [16]. TEAS has been used for perioperative anesthesia management and as part of postoperative recovery, which is useful for reducing intraoperative anesthesia medication [17], postoperative pain [18], postoperative nausea and vomiting [19], and postoperative recovery [20], and may be a promising treatment option for male infertility [21]. Currently, there has been no study on TEAS combined with PPI in the treatment of LPRD; thus, this trial has strong innovation and practical significance. This trial aims to evaluate the effectiveness and safety of this new treatment method for LPRD.

## Methods/design

### Study design

This prospective, randomized, parallel-designed clinical trial will be conducted in a tertiary hospital in Anhui, China. The trial protocol has been approved and reviewed by the Clinical Medical Research Ethics Committee of the First Affiliated Hospital of Anhui Medical University (PJ2021-04-20) and will be reported in accordance with the SPIRIT guidelines [22]. The corresponding author will be responsible for scientific supervision of the experiment. The study was registered with the Chinese Clinical Trial Registration Center (ChiCTR2100046755) on May 28, 2021.

### Study population

The study population will include patients diagnosed with LPRD in the Outpatient Department of Otorhinolaryngology Head and Neck Surgery of the First Affiliated Hospital of Anhui Medical University, People’s Republic of China. Two members of our research team will be responsible for recruiting potential trial participants. Written informed consent will be obtained by the clinical research coordinator at the outpatient department before randomization.

### Inclusion criteria

 ① Patients aged ≥ 18 years

② Patients with symptoms of globus sensation, continuous throat clearing, hoarseness, pronunciation fatigue, throat pain, chronic cough, and dyspnea for more than 6 weeks

③ Patients with RSI > 13 points and/or RFS > 7 points, with Dx-pH monitoring diagnosed as acid reflux

④ Patients who agree to sign an informed consent form and participate in clinical trials

### Exclusion criteria

 ① Patients with pharyngeal discomfort caused by bacterial or viral pharyngitis

② Patient with erosive gastroesophageal reflux, laryngeal cancer, esophageal cancer, gastric cancer, and other diseases revealed by upper gastrointestinal endoscopy or laryngoscopy

③ Patients with a history of esophageal or stomach surgery and neck radiotherapy

④ Patients with severe liver, kidney, and other organ dysfunctions and are unable to tolerate medications or allergic to medications

⑤ Patients receiving proton pump inhibitor therapy or other research drugs in the previous month

⑥ Patients who are pregnant or breastfeeding

⑦ Patients with RSI scale score (excluding the ninth item) < 10 points

⑧ Patients taking clopidogrel or warfarin

⑨ Patients contraindicated to undergo TEAS because of the following: skin rash or local infection or implantation of a pacemaker or defibrillator, electrode pads causing skin allergies or itching, acute diseases, infectious diseases, malignant tumors, cardiovascular and cerebrovascular diseases, and other malignant diseases

### Randomization and allocation concealment

We will randomize the patients according to the order of the random number table generated by the Statistical Package for the Social Sciences (SPSS) version 23.0 (IBM Corporation, New York, USA). Seventy participants will be divided into an intervention group and a control group at a ratio of 1:1. The statistician in charge of randomization will not participate in the evaluation, treatment, and result analysis of this study to ensure that the baseline RSI scores of each group are balanced. Those who will not be involved in opening the envelope in advance will put the random treatment allocation plan in a set of sealed envelopes. Each envelope must be numbered (to be able to identify all envelopes at the end of the study), opaque (to prevent transparency and visibility under strong light), and in other ways to prevent cheating. When randomizing a participant, his/her name and number on the next unopened envelope in front of the second staff member will be first recorded. Both staff members will sign the envelope, open it, and assign the grouping scheme contained therein to the participants and record this. Because it is difficult to design a placebo control for TEAS, blinding will not be used during the trial. Both the participants and outcome assessors will be aware of the grouping scheme. Statisticians who are blinded to group allocation will be responsible for analyzing the data.

### Interventions

For patients diagnosed with LPRD, we will conduct diet and lifestyle education, including quitting smoking and alcohol, low-fat diet, fasting 3 h before going to bed, and avoiding spicy fried, coffee, and carbonated drinks. The control group will receive conventional PPI acid suppression treatment, omeprazole 20 mg bid orally, and the intervention group will be treated with TEAS for 12 weeks on this basis. Because LPRD requires long-term treatment and takes into account the time barriers required for patients to frequently return to the hospital, most of the treatments are performed at home by the patients. Participants can easily identify whether the electrotherapy device is turned on and whether it is performing effective electrical stimulation. Thus, it is difficult to set blind methods in this experiment.

### Transcutaneous electrical acupoint stimulation

A household intermediate frequency pulse electrotherapy instrument (JF-ZP-YY01, Tianchang Jianfa Ziran Medical Products Co. Ltd, Chuzhou, China) with a pulse width of 280 us ± 10% will be used for TEAS in this trial. It has two 1.2 kHz ± 10% output channels and different stimulation intensities. The stimulation intensity can be adjusted according to each individual’s feeling. The stimulation intensity should be gradually increased to the maximum stimulation threshold without feeling pain or discomfort. The intermediate-frequency pulse electrotherapy instrument is easy to operate. The first operation is performed in the outpatient treatment room. Professional Chinese medicine physicians will guide the patient and observe whether there are adverse reactions during the first treatment. The participants will be instructed to record the treatment diary and record the operation time.

**Fig. 1:**
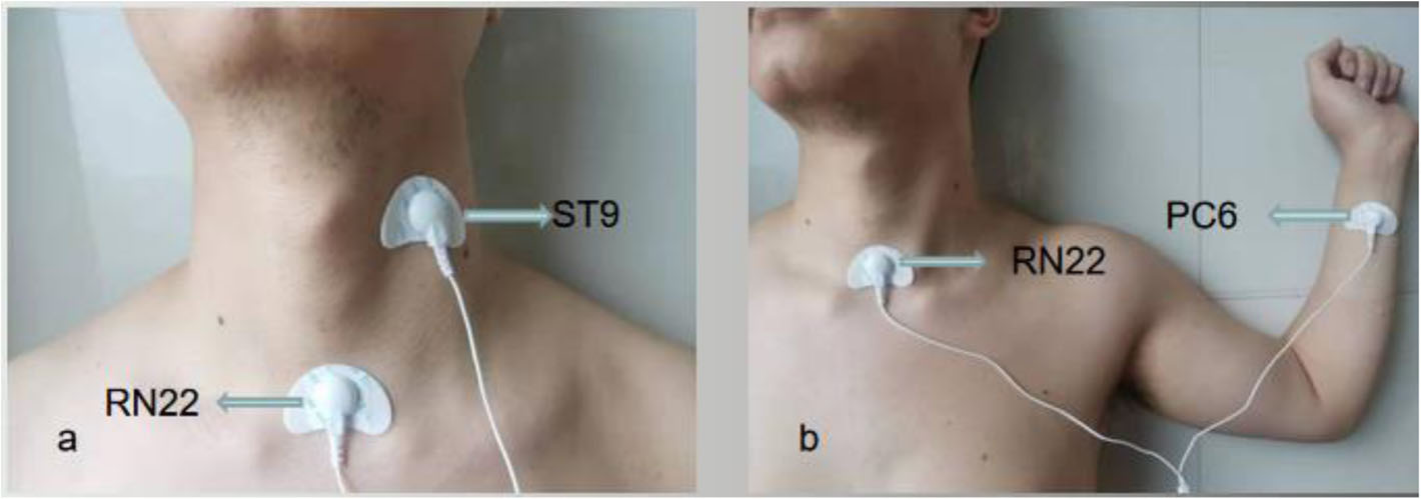
Locations of acupoints. **a** *Tiantu* (RN22) and *Renying* (ST9) on the left. **b** *Tiantu* (RN22) and *Neiguan* (PC6) on the left

According to the previous literature, we will use the following three acupoints for the treatment of LPRD [23–25]: *Tiantu* (RN22), *Renying* (ST9), and *Neiguan* (PC6) (Table 1 and Fig. 1).

**Table 1:** Locations of acupoints in intervention group

Acupoints	Locations
*Tiantu* (RN22)	Located on the neck, the current midline, in the center of the suprasternal fossa, between the left and right sternocleidomastoid muscles
*Renying* (ST9)	In the neck, next to the laryngeal junction, the front edge of the sternocleidomastoid muscle, the common carotid artery
*Neiguan* (PC6)	On the palm side of the forearm, two cun^a^ above the transverse crease of the wrist, between the palmar longus tendon and the flexor carpi radialis tendon

^a^1 cun (≈ 20 mm) is defined as the width of the interphalangeal joint of patient’s thumb

Traditional Chinese medicine believes that LPRD is a syndrome of deficiency of the essence and deficiency of yin. It is often based on qi deficiency and yin deficiency, with Qi stagnation, phlegm dampness, phlegm fire, cold, and heat as the standard. Disorders of the spleen, stomach, liver, and gallbladder are the main causes of this disease. The main principles of treatment are to regulate qi and relieve depression, resolve phlegm, reduce adversity, nourish yin and clear fire, and invigorate the spleen and stomach [26]. Wang et al. showed that acupuncture at RN22 and PC6 acupoints combined with esomeprazole tablets and that mosapride tablets is more effective than Western medicine alone [23]. Zhang et al. reported that acupuncture at the RN22 point combined with omeprazole can achieve satisfactory results in the treatment of reflux disease of the throat [24]. The therapeutic effect of acupoint injection at RN22 combined with electroacupuncture at the ST9 acupoint is better than that of oral drugs in the treatment of chronic pharyngitis [27].

The first group will consist of RN22 and ST9 acupoints on one side, and the second group will consist of RN22 and PC6 acupoints on one side. Each group will be treated for 15 min; after one side will be completed, the contralateral two groups will be treated. Therefore, one treatment will be 60 min in total, once a day, five times a week. The patient will be instructed not to increase or decrease the number of treatments.

Every patient in the intervention group will receive the instruction manual of the instrument and the detailed treatment plan. The treatment will be mainly performed at home and will be completed by the patient. The first treatment will be performed in the outpatient department of the Otolaryngology Department, guided and demonstrated by professional Chinese medicine physicians. In the initial treatment stage, the patient will be instructed to take photos and provide feedback to ensure that the patient chooses the correct acupoints. For patients with operational problems, we will arrange professionals to provide telephone and video guidance. In addition, we will record a video of the treatment steps to ensure that the patient understands the location of the acupoints and the treatment steps.

### Proton pump inhibitor

Both groups of patients will receive PPI treatment, one pill at a time, twice a day, orally 30–60 min before meals. PPI will be used as the control treatment method because it has been the most conventional empirical treatment for LPRD [1]. No evidence supports the superiority of one PPI over another for LPRD; therefore, we will use the most commonly used omeprazole. Omeprazole must be taken twice a day, because studies have shown that no PPI can suppress acid (pH > 4 in the stomach) for more than 16 h [28].

### Outcomes

#### Primary outcome

**Fig. 2:**
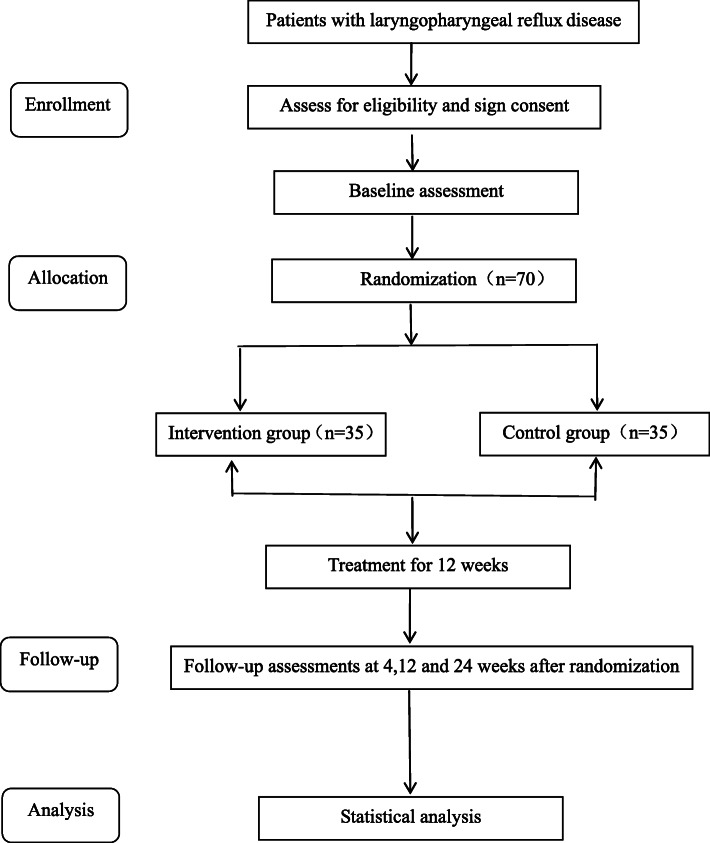
Study design

The RSI is a widely used scale for diagnosing and evaluating LPRD. It is a well-established patient self-report questionnaire and is a tool with publicly available data. The RSI scale contains 9 items, which are scored on a 6-point Likert scale (0–5), with a score of 0–45. The higher the score, the more severe the symptoms. RSI > 13 points will be considered possible patients with LPR [29]. RSI remains the “standard” in this field, although it has some shortcomings and limitations [30]. Therefore, we will select it as the primary outcome to assess the severity of reflux. Participants will be evaluated for changes in scale scores before the start of the experiment (after the informed consent will be obtained) and at 4, 12, and 24 weeks of the experiment (Fig. 2). The last of the nine items of the RSI is a complex GORD problem that includes heartburn, chest pain, indigestion, and acid reflux. Patients with dyspepsia may reach 5 points on the ninth item, which may be mistaken for patients with LPR. Referring to the report of a clinical trial, we will require that after the ninth item is removed, the score must be greater than or equal to 10 to be included [31]. Significant improvement will be defined statistically here, when the total score values will decrease by more than 50%. In addition, the valid rate will be defined as the proportion of patients showing significant and effective improvements.

#### Secondary outcomes

The reflux finding score (RFS) is a widely used scale [32]. According to the results of laryngoscopy, it assesses whether there are signs of vocal cord edema, granuloma in the larynx, posterior hyperplasia, mucosal hyperemia, or erythema. The total RFS score ranges from 0 to 29. Patients will be instructed to undergo laryngoscopy before treatment and 4 and 12 weeks after treatment to objectively assess the changes in reflux signs.

### Laryngopharyngeal Reflux-Health-related Quality of Life

Laryngopharyngeal Reflux-Health-related Quality of Life (LPR-HRQL) is a tool specially used to assess the quality of life of patients with throat symptoms, which can effectively and reliably assess the quality of life of patients with LPR [33, 34]. It has a total of 43 items divided into four areas and an overall impact category. The higher the score, the greater the impact of the disease on the patient’s quality of life, and the scale is significantly sensitive to changes [35].

### Visual analog scale for throat pain

RSI lacks an evaluation of throat pain, but throat pain is a common symptom in patients with LPR. Therefore, a VAS will be added to indicate changes in the patients before and after treatment. A score of 0 indicates no throat pain, and a score of 10 indicates extreme throat pain. Patients will be scored according to the severity of their symptoms.

### Dx-pH monitoring

The oropharyngeal pH test is an important test for the objective diagnosis of LPR. Some researchers compared the ability of oropharyngeal and esophageal pH monitoring to predict the response of patients with suspected LPR to PPI treatment, indicating that the oropharyngeal probe has a higher positive predictive power than esophageal measurement [36]. All patients will be required to undergo Dx-pH testing before enrollment to determine whether they have acid, alkaline, or mixed reflux. Only patients with acid reflux will be included. Because PPI has a great effect on acidic and mixed reflux, it can alkalize the gas and liquid of reflux and reduce the activity of pepsin, but it has little effect on alkaline reflux [37]. In previous studies, because of the time and economic cost of pH testing, most patients are unwilling to perform pH monitoring again after treatment. Therefore, we will consider pH detection as a secondary outcome.

**Fig. 3:**
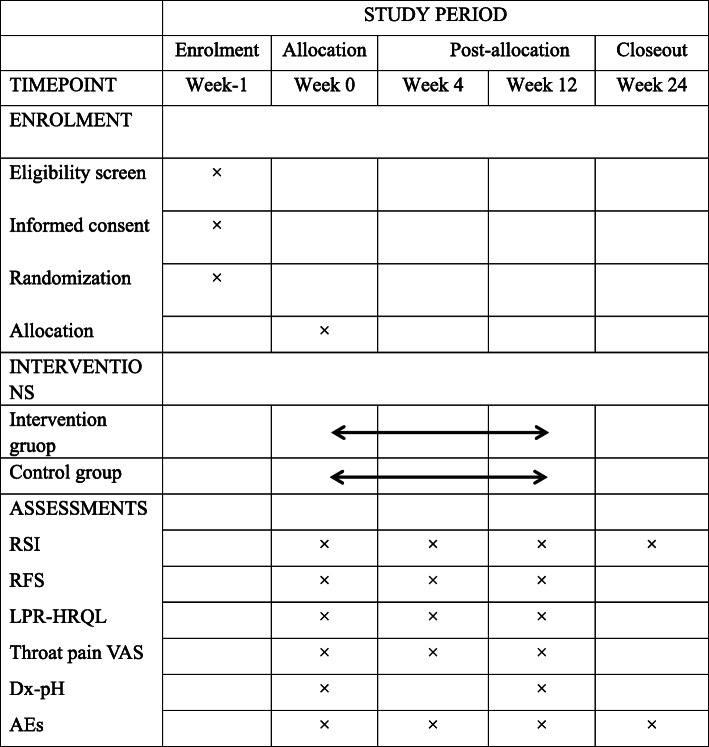
Schedule of enrolment, intervention, and assessment (SPIRIT) figure

All outcomes will be measured at the four time points of the study. Dx-pH monitoring will be performed at baseline and 12 weeks. LPR-HRQL score, throat pain VAS score, and RFS will be assessed at baseline, 4, and 12 weeks. RSI will be evaluated at baseline, and 4, 12, and 24 weeks. The time schedule for the inclusion of participants, intervention, and evaluation is shown in Fig. 3.

### Adverse events

Adverse events (AEs) will be reported to the data monitoring ethics committee, and experts will classify AEs as treatment-related or non-treatment-related based on their potential association with TEAS or PPI within 24 h after the occurrence. If a severe AE will occur, the participant will be required to withdraw from the trial immediately and seek medical help at the nearest general hospital. There will be no anticipated harm or compensation for trial participation.

### Quality assurance and quality control

To ensure the quality of the study, otolaryngology experts in Chinese medicine, Western medicine, and statisticians will review and revise the study protocol prior to the start of the experiment. Other standardized procedures for research operations, including details such as filling out questionnaires, recruiting participants, treatment interventions, AE evaluation, and data management, have also developed uniform standards. Any deviations from the protocol will be fully documented using a breach report form. We will update the protocol in the clinical trial registry in the event of protocol modifications. The Project Management Group will meet every 3 months to review the trial conduct. The Clinical Medical Research Ethics Committee of the First Affiliated Hospital of Anhui Medical University will meet every half year to review conduct throughout the trial period. The clinical research assistant will periodically review the data to ensure the authenticity, timeliness, and data quality of the data collection. All data will be collected in the form of paper + electronic questionnaires, finally transferred to an Excel spreadsheet, and stored for at least 5 years after the trial results will be published. Therefore, all data collected during the research process will be kept strictly confidential and used only by researchers. The patient’s private information, including name and phone number, will be strictly protected and will never be allowed to be disclosed. If reviewers or readers have any questions about our published data, they can contact the corresponding author to obtain the data. Researchers will make detailed treatment plan descriptions, acupoint pictures, videos, and TEAS treatment procedures and distribute them to each participant. Researchers will ensure that the participants comply with the intervention plan. A specially established communication team will guide participants in their interventions and will be available to answer their questions. All treatments will be free of charge to participants, and for those who will discontinue or deviate from the intervention protocols, we will keep the data we have collected and record the reasons for quitting. Participants will be registered with a phone number and address for further contact if they miss the follow-up time.

### Statistical methods

#### Sample size

According to the previous literature on the use of PPI therapy in the Chinese population [38], after 3 months of LPRD treatment, the RSI score was approximately 11.7 points, and the standard deviation was approximately 6. We will estimate that the RSI score of the intervention group will be 4.5 points higher than that of the control group after treatment. Comparing two independent samples using a two-tailed *t*-test with an alpha value of 0.05% and 80% power, a sample size of 29 patients will be obtained. Considering a dropout rate of 10%, 33 patients will be recruited for each group. In actual recruitment, we will plan to recruit 35 participants for the intervention and control groups. We will plan to recruit sufficient participants by posting posters in ENT clinics for 4 months.

#### Statistical analyses

The measurement data that conform to the normal distribution will be represented by the mean ± standard deviation, and the measurement data that do not conform to the normal distribution will be represented by M (P25, P75). The analysis will be performed based on intention-to-treat and will be performed using SPSS version 23.0. A bilateral *P* value of less than 0.05 will be considered significant. Continuous variables will be compared using the t-test or Mann–Whitney *U* test. The chi-squared test or Fisher’s exact test will be used to compare the qualitative data.

## Discussion

This study protocol introduces the design of a randomized controlled trial to clarify the effectiveness and safety of TEAS combined with PPI in the treatment of LPRD. To the best of our knowledge, there are no studies using TEAS to treat LPRD. Thus, this trial is significantly innovative.

In recent decades, the standard treatment for LPR includes taking PPIs before breakfast and dinner. However, compared with placebo, the effectiveness of PPI treatment is weak [14]. Moreover, long-term use of PPIs may cause osteoporosis and endocrine and kidney dysfunctions [37]. Therefore, the use of PPI alone is not effective in the treatment of LPRD, and it is necessary to explore the treatment of TEAS combined with PPIs.

Compared with traditional acupuncture and needle-based electrical stimulation, TEAS has a comparative advantage due to its noninvasive characteristics and the possibility of continuous and multiple stimulations. LPRD has brought a huge economic burden and has attracted increasing attention. The use of an electrotherapy instrument for home treatment of TEAS is feasible, innovative, and sustainable, with good patient compliance.

One limitation of this study is the failure to set the blinding methods. Second, despite quality assurance and quality control, considering that patients’ TEAS treatment is mainly performed at home, it is difficult to ensure that the participants’ treatment methods are completely correct. This study will validate the efficacy and safety of TEAS combined with PPI in the management of LPRD and offers a new and promising therapeutic modality for treating patients with LPRD. Our results will be published in peer review journals in the form of articles.

## Trial status

Protocol: version 2.0, July 10, 2021. The first patient was recruited on June 1, 2021, and the last patient is expected to be recruited on October 1, 2021. This protocol was submitted prior to the recruitment of the 70 patients.

## Supplementary Information

**Additional file 1.** Completed Standard Protocol Items: Recommendation for Interventional Trials (SPIRIT) 2013 checklist: items addressed in this clinical trial protocol.

## Data Availability

The datasets analyzed during the current study are available from the corresponding author upon reasonable request.

## Abbreviations

LPR: Laryngopharyngeal reflux
LPRD: Laryngopharyngeal reflux disease
RSI: Reflux Symptom Index
GORD: Gastroesophageal reflux disease
PPI: Proton pump inhibitor
TEAS: Transcutaneous electrical acupoint stimulation
RFS: Reflux finding score
VAS: Visual analog scale
AEs: Adverse events

## References

[1] Koufman JA, Aviv JE, Casiano RR, Shaw GY (2016). Laryngopharyngeal reflux: position statement of the committee on speech, voice, and swallowing disorders of the American Academy of Otolaryngology-Head and Neck Surgery. Otolaryngol Head Neck Surg.

[2] Lechien JR, Akst LM, Hamdan AL, Schindler A, Karkos PD, Barillari MR, Calvo-Henriquez C, Crevier-Buchman L, Finck C, Eun YG, Saussez S, Vaezi MF (2019). Evaluation and management of laryngopharyngeal reflux disease: state of the art review. Otolaryngol Head Neck Surg.

[3] Koufman JA (1991). The otolaryngologic manifestations of gastroesophageal reflux disease (GERD): a clinical investigation of 225 patients using ambulatory 24-hour pH monitoring and an experimental investigation of the role of acid and pepsin in the development of laryngeal injury. Laryngoscope.

[4] Tasker A, Dettmar PW, Panetti M, Koufman JA, Birchall JP, Pearson JP. Reflux of gastric juice and glue ear in children. Lancet. 2002;359(9305):493. 10.1016/S0140-6736(02)07665-1. 10.1016/S0140-6736(02)07665-1 11853797

[5] Arruda Henry MA, Martins RH, Lerco MM, Carvalho LR, Lamônica-Garcia VC (2011). Gastroesophageal reflux disease and vocal disturbances. Arq Gastroenterol.

[6] Vaezi MF, Hicks DM, Abelson TI, Richter JE (2003). Laryngeal signs and symptoms and gastroesophageal reflux disease (GERD): a critical assessment of cause and effect association. Clin Gastroenterol Hepatol.

[7] Xiao S, Li J, Zheng H, Yan Y, Li X, Zhang L, Lv Q, Zhang J, Zeng L, Gao X, Chen X, Yang H, Zhao C, Zhang J, Lu H, Luo X, Wang G, Yi H, Ye J, Lin Z, Tian L, Zhang J, Chen T, Yu A, Liu Z, Ren X, Yang X, Zhang S, Cui X, Li G, Wan G, Lin C, Chen H, Deng A, Tang X, Zhang Q, Tao Z, Shi L, Zhou J, Qin G, Zhuang P, Huangfu H, Yang J, Zhou G, Li H, Wu W, Li J, Li S, Lou G, Fang H, Ma J, Shan C, Zhou X, Tang L, Zhou F, Fan Y, Zhang Y, Li Y, Li M, Dou C, Chen Z, Lei G, Li J, Gao Z, Huang Y, Ma X, Liu Z, Liang G, He J, Zhao H, Song B, Chen M, Yang X, Ma Z, Ren J (2020). An epidemiological survey of laryngopharyngeal reflux disease at the otorhinolaryngology-head and neck surgery clinics in China. Eur Arch Otorhinolaryngol.

[8] Lam P, Wei WI, Hui Y, Ho WK (2006). Prevalence of pH-documented laryngopharyngeal reflux in Chinese patients with clinically suspected reflux laryngitis. Am J Otolaryngol.

[9] Spantideas N, Drosou E, Bougea A, Assimakopoulos D (2015). Laryngopharyngeal reflux disease in the Greek general population, prevalence and risk factors. BMC Ear Nose Throat Disord.

[10] Kamani T, Penney S, Mitra I, Pothula V (2012). The prevalence of laryngopharyngeal reflux in the English population. Eur Arch Otorhinolaryngol.

[11] Koufman JA, Amin MR, Panetti M (2000). Prevalence of reflux in 113 consecutive patients with laryngeal and voice disorders. Otolaryngol Head Neck Surg.

[12] Altman KW, Stephens RM, Lyttle CS, Weiss KB (2005). Changing impact of gastroesophageal reflux in medical and otolaryngology practice. Laryngoscope.

[13] Francis DO, Rymer JA, Slaughter JC, Choksi Y, Jiramongkolchai P, Ogbeide E, Tran C, Goutte M, Garrett GC, Hagaman D, Vaezi MF (2013). High economic burden of caring for patients with suspected extraesophageal reflux. Am J Gastroenterol.

[14] Lechien JR, Saussez S, Schindler A, Karkos PD, Hamdan AL, Harmegnies B, de Marrez LG, Finck C, Journe F, Paesmans M, Vaezi MF (2019). Clinical outcomes of laryngopharyngeal reflux treatment: a systematic review and meta-analysis. Laryngoscope.

[15] Guo H, Ma H, Wang J (2016). Proton pump inhibitor therapy for the treatment of laryngopharyngeal reflux: a meta-analysis of randomized controlled trials. J Clin Gastroenterol.

[16] Li H, Wu C, Yan C, Zhao S, Yang S, Liu P, Liu X, Wang M, Wang X (2019). Cardioprotective effect of transcutaneous electrical acupuncture point stimulation on perioperative elderly patients with coronary heart disease: a prospective, randomized, controlled clinical trial. Clin Interv Aging.

[17] Wetzel B, Pavlovic D, Kuse R, Gibb A, Merk H, Lehmann C, Wendt M, Usichenko TI (2011). The effect of auricular acupuncture on fentanyl requirement during hip arthroplasty: a randomized controlled trial. Clin J Pain.

[18] Sahmeddini MA, Farbood A, Ghafaripuor S (2010). Electro-acupuncture for pain relief after nasal septoplasty: a randomized controlled study. J Altern Complement Med.

[19] Liu Y, Tang WPY, Gong S, Chan CWH (2017). A systematic review and meta-analysis of acupressure for postoperative gastrointestinal symptoms among abdominal surgery patients. Am J Chin Med.

[20] Yao Y (2015). Transcutaneous electrical acupoint stimulation improves the postoperative quality of recovery and analgesia after gynecological laparoscopic surgery: a randomized controlled trial. Evid Based Complement Alternat Med.

[21] Jin ZR, Fang D, Liu BH, Cai J, Tang WH, Jiang H, Xing GG (2021). Roles of CatSper channels in the pathogenesis of asthenozoospermia and the therapeutic effects of acupuncture-like treatment on asthenozoospermia. Theranostics.

[22] Chan A-W, Tetzlaff JM, Gøtzsche PC, Altman DG, Mann H, Berlin J, Dickersin K, Hróbjartsson A, Schulz KF, Parulekar WR, Krleža-Jerić K, Laupacis A, Moher D (2013). SPIRIT 2013 Explanation and Elaboration: Guidance for protocols of clinical trials. BMJ.

[23] Wang YY, Xu W, Wang X, Gao W, He L, Su Y, Du X (2020). Clinical study of laryngopharyngeal reflux treated with acupuncture on the base of theory of the ascending and the descending of qi. Zhongguo Zhen Jiu.

[24] Zhang T (2017). Clinical observation of acupuncture on Tiantu acupoint combined with western medicine in the treatment of laryngopharyngeal reflux disease. Shanghai J Tradit Chin Med.

[25] Qu ZY, Sun HB (2016). Treating laryngopharyngeal reflux disease from treating flaccidity. Henan Tradit Chin Med.

[26] Li YY (2019). Thinking of TCM syndrome differentiation of laryngopharyngeal reflux disease. Chin J Integr Tradit Chin Western Med Otorhinolaryngol.

[27] Wang QQ, Chen HL (2005). Therapeutic effect of acupoint-injection combined with electroacupuncture on chronic pharyngitis. Zhongguo Zhen Jiu.

[28] Rees CJ, Belafsky PC (2008). Laryngopharyngeal reflux: current concepts in pathophysiology, diagnosis, and treatment. Int J Speech Lang Pathol..

[29] Belafsky PC, Postma GN, Koufman JA (2002). Validity and Reliability of the Reflux Symptom Index (RSI). J Voice.

[30] Papakonstantinou L, Leslie P, Gray J, Chadwick T, Hudson M, Wilson JA (2009). Laryngopharyngeal reflux: a prospective analysis of a 34 item symptom questionnaire. Clin Otolaryngol.

[31] O'Hara J (2021). Use of proton pump inhibitors to treat persistent throat symptoms: multicentre, double blind, randomised, placebo controlled trial. BMJ.

[32] Belafsky PC, Postma GN, Koufman JA (2001). The validity and reliability of the reflux finding score (RFS). Laryngoscope.

[33] Lenderking WR, Hillson E, Crawley JA, Moore D, Berzon R, Pashos CL (2003). The clinical characteristics and impact of laryngopharyngeal reflux disease on health-related quality of life. Value Health.

[34] Carrau RL, Khidr A, Gold KF, Crawley JA, Hillson EM, Koufman JA, Pashos CL (2005). Validation of a quality-of-life instrument for laryngopharyngeal reflux. Arch Otolaryngol Head Neck Surg.

[35] Vaezi MF, Richter JE, Stasney CR, Spiegel JR, Iannuzzi RA, Crawley JA, Hwang C, Sostek MB, Shaker R (2006). Treatment of chronic posterior laryngitis with esomeprazole. Laryngoscope.

[36] Becker V, Graf S, Schlag C, Schuster T, Feussner H, Schmid RM, Bajbouj M (2012). First agreement analysis and day-to-day comparison of pharyngeal pH monitoring with pH/impedance monitoring in patients with suspected laryngopharyngeal reflux. J Gastrointest Surg.

[37] Savarino V, Di Mario F, Scarpignato C (2009). Proton pump inhibitors in GORD: an overview of their pharmacology, efficacy and safety. Pharmacol Res.

[38] Lam PK (2010). Rabeprazole is effective in treating laryngopharyngeal reflux in a randomized placebo-controlled trial. Clin Gastroenterol Hepatol.

